# Exercise and nutrition for head and neck cancer patients: a patient oriented, clinic-supported randomized controlled trial

**DOI:** 10.1186/1471-2407-12-446

**Published:** 2012-10-02

**Authors:** Lauren C Capozzi, Harold Lau, Raylene A Reimer, Margaret McNeely, Janine Giese-Davis, S Nicole Culos-Reed

**Affiliations:** 1 Faculty of Kinesiology, University of Calgary, KNB 2229 2500 University Drive NW, Calgary, Alberta T2N 1N4, Canada; 2 Department of Oncology, Division of Psychosocial Oncology, University of Calgary, Calgary, Canada; 3 Alberta Health Services—Cancer Care, Tom Baker Cancer Centre, Psychosocial Resources, Calgary, Canada; 4 Department of Oncology, Alberta Health Services, Calgary, Canada; 5 Faculty of Rehabilitation Medicine, University of Alberta & Cross Cancer Institute, Edmonton, Canada

**Keywords:** Head/neck cancer, Exercise, Progressive strength training, Quality of life, Cancer, Randomized controlled trial, Nutrition, Lifestyle intervention

## Abstract

**Background:**

Research on physical activity and nutrition interventions aimed at positively impacting symptom management, treatment-related recovery and quality of life has largely excluded head and neck (HN) cancer populations. This translates into a lack of clinical programming available for these patient populations. HN cancer patients deal with severe weight loss, with more than 70% attributed to lean muscle wasting, leading to extended recovery times, decreased quality of life (QoL), and impaired physical functioning. To date, interventions to address body composition issues have focused solely on diet, despite findings that nutritional therapy alone is insufficient to mitigate changes. A combined physical activity and nutrition intervention, that also incorporates important educational components known to positively impact behaviour change, is warranted for this population. Our pilot work suggests that there is large patient demand and clinic support from the health care professionals for a comprehensive program.

**Methods/Design:**

Therefore, the purpose of the present study is to examine the impact and timing of a 12-week PA and nutrition intervention (either during or following treatment) for HN cancer patients on body composition, recovery, serum inflammatory markers and quality of life. In addition, we will examine the impact of a 12-week maintenance program, delivered immediately following the intervention, on adherence, patient-reported outcomes (i.e., management of both physical and psychosocial treatment-related symptoms and side-effects), as well as return to work.

**Discussion:**

This research will facilitate advancements in patient wellness, survivorship, and autonomy, and carve the path for a physical-activity and wellness-education model that can be implemented in other cancer centers.

**Trial registration:**

Current Controlled Trials NCT01681654

## Background

Head and neck (HN) cancers include cancers of the lip, oral cavity, oropharynx, hypopharynx, tonsil, salivary glands, nasopharynx, nose paranasal sinus, and middle ear [1]. They are the sixth most common cancer worldwide [2]. HN cancers account for approximately 3% of all cancers in the United States and are nearly twice as common among men as they are among women [1]. In 2011, approximately 52,000 people in the United States were diagnosed with HN cancers and over 5000 in Canada, and the 5-year survival rate is currently at 57% [1,3]. Advances in medical detection and interventions are helping to increase 5-year overall cancer survival rates, leading to an ever-growing number of cancer survivors [4]. Since the long-lasting effects of the disease and treatments may impact all survivors, the focus on survivorship care and patient outcomes is paramount [5].

There is persuasive evidence to suggest that two lifestyle behaviors, physical activity (PA) and nutrition are necessary components in cancer survivorship programming [6]. PA has been shown to enhance patient functional capacity, physical functioning, and body composition, as well as facilitate the management of treatment related symptoms and side-effects including fatigue, and nausea [7-11]. Nutritional counseling has been found to improve dietary intake in patients who face increased risk of malnutrition, thereby improving outcomes and timely healing [12,13]. It is well established that PA and nutrition interventions also promote improved quality of life (QoL) in cancer survivors, regardless of tumour site. To date, the vast majority of lifestyle interventions have focused their attention on breast cancer [12,14-16]. This has left other cancer populations drastically underserved, including HN cancer survivors, who are at significant risk of long-term disability [17].

While HN cancer patients face issues similar to other cancer survivor populations (i.e., fatigue, distress, physical functioning), these populations also experience further physical and psychological burden into survivorship, affecting overall QoL status, rehabilitation, and return to work [17-21]. HN cancer patients deal with severe weight loss, with upwards of 70% attributed to lean muscle wasting, leading to decreased physical functioning with regards to locomotion, strength and respiration after treatment [19,22]. This cancer-related skeletal muscle wasting, or cachexia, exists due to imbalances between muscle protein synthesis and degradation, and is unique from starvation in the sense that it cannot be reversed or treated solely with nutritional supplementation [23]. Cachexia is defined as an unintentional weight loss of at least 5% premorbid weight occurring over 3 to 6 months and will be the main cause of death for 20-40% of HN cancer patients, a group especially challenged with this syndrome [23,24].

Current interventions are largely focused on nutrition, despite findings indicating that decreased food intake is probably not directly related to this muscle wasting, and that dietary intervention alone may increase fat mass without affecting muscle tissue [25]. Couch et al. (2007) also reported that although nutritional therapy is necessary, alone it might be insufficient, prompting additional or complementary treatment options [24]. At present there is a lack of complementary treatment options to help sustain or rebuild this wasted muscle, yet since cachexia is a muscle wasting disease, interventions targeted at increasing protein anabolism and decreasing catabolism are warranted [26]. Strength training and exercise hold great potential due to the associated increases in lean muscle mass and improvements in body composition, which have been seen in other cancer populations [8,27]. Currently, there are very few clinical trials that examine the impact of strength training in combination with a nutritional intervention for cachexia. Besides the negative impact on overall health and survival, this muscle wasting syndrome is associated with substantial mental and emotional fatigue, and significantly decreased QoL, particularly in scores of emotional, physical and social functioning categories [18,23,24,28,29]. Exercise and diet have been found to enhance QoL in HN survivors and help manage fatigue, but the specific benefits of a lifestyle intervention during treatment has yet to be investigated, and the question pertaining to the appropriate time of intervention, during treatment or following treatment, has yet to be answered [17,30].

The purpose of this proposed research is to examine the immediate and long term physical and psychosocial benefits of a clinic-supported physical activity and nutrition program during and after treatment for HN cancer survivors, and to investigate the clinical implications associated with increasing the promotion of healthy lifestyle behaviors for this population. This research will address the pressing need for clinic-supported HN cancer lifestyle interventions that effectively demonstrate physical and psychosocial patient benefit.

Specifically, this study is designed to examine the potential benefits of a lifestyle (PA, nutrition, and health education) intervention. Specific objectives include the following:

1. Whether a lifestyle intervention will improve outcomes, including physiological (muscle mass maintenance), fitness, psychological (QoL and depression), smoking status, inflammatory markers, and functional (return to work).

2. Whether it is optimal, in terms of outcomes, to deliver the program during or after treatment completion.

3. Whether a survivorship care plan (SCP) maintenance program improves long-term healthy lifestyle adherence.

Without exploration into these questions, HN cancer care will continue to only partially serve patients in their quest towards healthy cancer recovery, strong physical functioning ability, improved QoL, and timely return to work. These studies will provide concrete evidence for a clinic-supported lifestyle intervention and maintenance program as a means to mediate these currently unaddressed factors and improve functioning and psychosocial outcomes.

The study hypotheses directly reflect the aims. It is hypothesized that patients who are randomized to the 12-week lifestyle intervention at treatment start will experience improved symptom management throughout treatment compared to controls (delayed intervention) as evaluated by: a) decreased loss of lean body mass, improved physical functioning and fitness outcomes, improved Karnofsky Performance scores and decreased levels of serum inflammatory markers b) improved patient sense of wellbeing and feelings of control and therefore overall reported QoL. It is also hypothesized that patients who are randomized to the 12-week lifestyle intervention at treatment start (immediate intervention) will experience improved symptom management following treatment compared to delayed intervention patients who begin the 12-week intervention following treatment. Improved symptom management will be evaluated following intervention by: a) decreased loss of lean body mass, improved physical functioning and fitness outcomes, improved Karnofsky Performance scores and decreased levels of serum inflammatory markers as well as the associated b) improvement in patient sense of wellbeing, feelings of control, and overall reported QoL score.

## Methods/Design

### Study design

**Figure 1:**
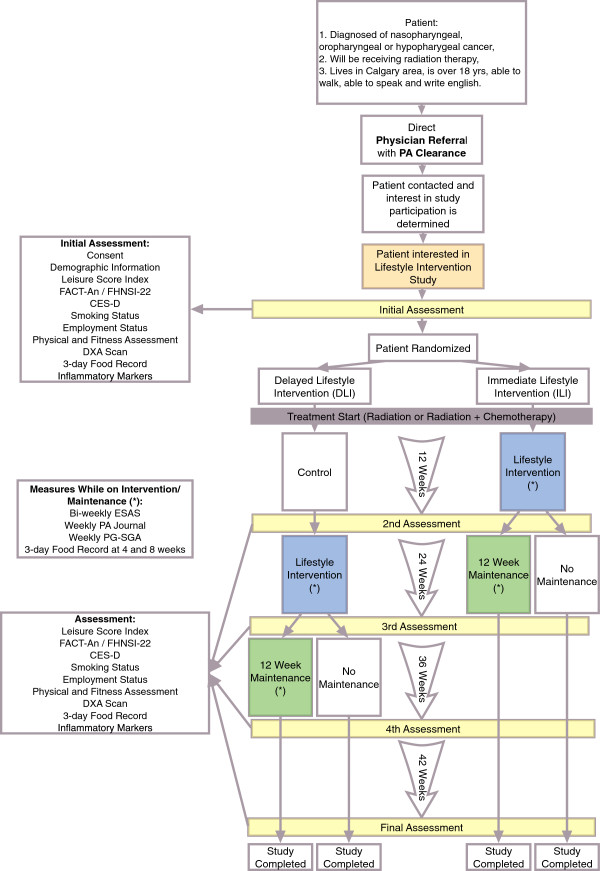
Study design protocol.

This study will be a randomized controlled trial, with an Immediate Lifestyle Intervention (ILI) group and a Delayed Lifestyle Intervention (DLI) group. The DLI group will act as the control group during the first 12-week phase. Flow through the study is portrayed in Figure 1. Research ethics approval to conduct this study was obtained through the Conjoint Health Research Ethics Board of the Faculties of Medicine, Nursing and Kinesiology, at the University of Calgary (Ethics ID: E-24446).

#### Treatment arms

Once enrolled, the participant will be randomized to either begin the intervention at the time of treatment initiation (ILI, n = 40), or to begin the intervention following treatment completion (DLI, n = 40). Patients will be recruited, cleared for participation, receive an initial assessment, and then be randomized into either the ILI group or the DLI group. Participants in the ILI group will receive an individualized exercise and dietary program based on their exercise assessment and dual energy x-ray absorptiometry (DXA) scan results, will attend twice weekly group exercise classes, and perform their individualized at-home program an additional two times per week. In addition, participants will be required to attend six education sessions during the 12-week intervention. The DLI group will receive this intervention upon their treatment completion, at approximately 12-weeks post-randomization.

#### Physical activity intervention

Participants will attend twice weekly exercise classes, for 12 weeks, at the Thrive Centre or Tom Baker Cancer Centre Holy Cross location to practice their program in a supported group-based environment. In addition, participants will receive equipment to perform the program at least two days per week at home. The focus of the PA program will be progressive strength training, helping to rebuild lost muscle mass and prevent further protein degradation. Participants will be asked to perform 8-10 prescribed low-intensity exercises four times per week, conducting approximately 2 sets of 8-10 repetitions. During this time, participants will be asked to record daily activity in a PA log.

#### Dietary intervention

Patients will be screened weekly using the Patient Generated-Subjective Global Assessment tool (PG-SGA) during treatment and will complete a 3-day dietary record at baseline, four weeks, eight weeks, and then at each assessment point thereafter [31,32]. This information will be scored and a dietitian will review and provide appropriate feedback at weekly dietary counseling sessions.

#### Educational intervention

The educational seminars will focus on providing both nutrition and PA information that will aid in the preparation for behavior change. Specifically, weekly education classes addressing the impact of lifestyle choices on treatment-related symptoms and outcomes, teaching behavior change techniques (goal setting, journaling etc.), and providing time for supportive discussion will be held immediately after an exercise session one time/week.

#### Maintenance intervention

Following the initial intervention, participants in both the immediate and delayed intervention arms will be randomized (20:20 in each group) to either a control (no maintenance program other than access to the workout center) or a 12-week maintenance program immediately following the intervention, consisting of an SCP with both exercise and dietary prescriptions, weekly group-based PA classes, and ongoing monthly education classes.

### Study population

The intervention participants will consist of HN patients who have been newly diagnosed and who have not yet started treatment. Patients will be eligible to participate if they: 1) are over 18 years of age 2) have received a diagnosis of nasopharyngeal, oropharyngeal or hypopharygeal cancer, 3) are scheduled to receive radiation treatment, 4) are able to walk without assistance, 5) have received clearance for exercise from their treating oncologist, 6) live in the Calgary area 7) can speak and write English, and 8) are interested in participating in the program. Reasons for exclusion will be documented as feasibility outcomes of the trial.

### Recruitment

Participants will be recruited through the HN clinic at the Tom Baker Cancer Centre, with direct referral and clearance for exercise from a physician. Once the participant is referred, they will be contacted to discuss the study details and, if interested and meet the inclusion criteria, will receive an appointment time for an initial assessment. At this time, participants will complete a written, informed consent before participation in the study.

### Measures

The assessments will consist of a battery of patient-reported instruments to assess demographics, medical information, PA and dietary behavior, QoL, depression, smoking status as well as a physical fitness assessment and a comprehensive body composition assessment. Inflammatory markers, including TNF, IL-6, IL-1, IL-8 and C-reactive protein will be assessed in serum. A chart review will also be conducted to assess diagnosis, treatment plan and outcomes. The assessment will take approximately 1.5 hours to complete and will occur at baseline (time of diagnosis), 3, 6, 9, and 12-months post diagnosis.

### Health and lifestyle questionnaires

#### Demographics

Demographics will be collected by self-report including gender, age, ethnicity, marital status, education, employment status, return to work status and annual income. Medical variables, abstracted from chart reviews by study staff, will include specific diagnosis (type, stage, initial or recurrence), time since diagnosis, and current treatment.

### Physical activity behaviour

PA will be assessed using Godin’s leisure score index (LSI) of the GLTEQ (Godin Leisure Time Exercise Questionnaire) [33]. The LSI is a 3-item measure assessing the frequency and duration of mild, moderate and strenuous bouts of exercise performed during free time in a typical week. In this study, participants will be asked to recall their exercise levels over the past month. The LSI has been successfully used with adult cancer patients and survivors, and has good reliability and validity as indicated by an independent evaluation to nine other self-report measures of exercise [34-36].

### Patient-reported outcomes

#### Quality of life

QoL will be assessed using the Functional Assessment of Cancer Therapy- Anemia module (FACT-AN), and the NCCN-FACT Fact Head/Neck Symptom Index-22 (FHNSI-22). These tools examine the 5 major subsets of well-being for cancer survivors, including physical well-being, social/family well-being, emotional well-being, functional well-being, and additional concerns specific to HN cancer survivors. Based on these scales, the Trial Outcome Index (TOI) will be calculated, providing a final score based on the sum of the subscales.

#### Depression

Depression will be assessed using the Center for Epidemiological Studies on Depression Scale (CES-D). This 20-item questionnaire screens for self-reported depressive symptoms [37]. It has shown excellent internal consistency in the cancer population with perfect sensitivity and strong specificity in a study of 33 cancer patients [38].

#### Smoking history

Smoking history will be assessed by a self-report questionnaire. This questionnaire will classify patients as non-smokers, former smokers and current smokers.

#### Weekly PA logs

Participants will complete daily PA logs. These logs will highlight daily activities, the intensity of those activities (1-10 Rated Perceived Exertion Scale), and the duration of those activities.

#### Biweekly Edmonton symptom assessment system (ESAS)

Participants will complete the ESAS bi-weekly, before and after class. The ESAS is a valid and reliable assessment tool to evaluate the nine more common symptoms experienced by cancer patients [39].

#### Diet behaviour

Diet will be assessed using a 3-day Diet Record once per month, and the PG-SGA will be completed once per week. The 3-Day Diet Record is said to be the most accurate for mean macronutrient content and appropriate for use in studies where subjects may consume a wide variety of foods [40,41]. Participants are instructed to record their daily consumption, including alcohol intake, over a period of three days, one of which must be a weekend day. Written instructions and a sample entry are provided to increase accuracy of the daily record. The PG-SGA assessment tool has been show to improve treatment outcomes, decrease side-effects, and improve weight-management in cancer patients, and therefore will be used weekly to assess and identify malnutrition among patients [31,32].

### Health-related fitness assessments

#### Body composition

Standing height and weight will be measured on a Heath Carter balance beam scale. Participants will be weighed weekly during active treatment and during the lifestyle intervention and maintenance phase. Weight will also be tracked at each of the 5 assessments. Waist circumference will be measured with an anthropometric tape measure at the top of the iliac crest and hip circumference will be measured at the greatest girth of the gluteus with the participant standing erect [42]. Waist to hip ratio and body mass index (kg × m^-2^) will be calculated [43]. A DXA scan (Hologic QDR 4500; Hologic Inc, Bedford, MA) will be performed a total of five times, at each assessment. Hologic QDR software will be used to determine lean mass, fat mass and bone mineral density. The DXA scans will be done at the Human Performance Laboratory at the University of Calgary.

#### Cardiorespiratory fitness

Although aerobic fitness is not the specific focus of the exercise intervention, the resistance training may likely have ancillary impact on cardiorespiratory fitness. While a submaximal cardiorespiratory test would be the standard, due to the limitations in this patient population, a functional measure of aerobic fitness was deemed to have greater value, as well as be more reflective of the training focus in the intervention. Resting heart rate will be measured using a heart rate monitor (Polar) and resting blood pressure (mmHg) will be measured in duplicate on the left arm using a sphygmomanometer and stethoscope using standardized procedures [43]. Rating of perceived exertion (Borg scale) will be completed immediately prior to and immediately after completion of the functional aerobic capacity test. The six-minute walk test (6MWT) will be used to assess changes in functional aerobic capacity. Using the standardized protocol, participants will be asked to walk as far as they can around a 400-meter track for six minutes [43]. The point reached at 6 minutes will be marked and measured to the nearest 0.5 meter. Post-exercise HR and BP will be measured one minute after a standard cool-down and after sitting quietly for five minutes before dismissal from the fitness-testing laboratory.

#### Musculoskeletal fitness

Muscular strength will be assessed using a combined grip strength of the right and left hands using a hand dynamometer. A sum will be determined in kilograms from the best score of 2 trials recorded for each hand according to the CPAFLA protocol [43]. Lower body strength will be assessed using a 30-second sit to stand test. The number of times participants can stand from a seated position in 30-second will be examined [44].

#### Flexibility

Flexibility will be assessed by a trunk forward flexion sit-and-reach test using a Wells-Dillon flexometer. The test will follow a standard protocol, with two trials allowed and the highest score to the nearest 0.5 cm recorded [43].

#### Balance

Balance will be assessed using a static balance test. The test requires the participant to balance on one foot and then the other as long as they can (length of time) while standing on a 2.54 by 2.54 by 30.5 cm base using a standardized protocol, reported by Fleishman [45].

### Measures of physical functioning

#### Participant performance score

The Karnofsky Performance Score (KPS) will be used to measure the participant’s general ability to accomplish tasks of daily-living and overall well-being. The Score is used to reflect the patient’s performance or function and ability to work, and is practical, objective, and has been validated and used for the past 50 years [46].

### Blood data collection

#### Inflammatory markers

Inflammatory factors will be evaluated as they are associated with cancer cachexia and muscle wasting and may be modified by exercise [47,48]. An overnight fasted blood draw will be collected at baseline, 3 months post diagnosis, 6 months post diagnosis, 9 months post diagnosis, and 12 months post diagnosis. Once further funding is secured, serum inflammatory cytokine concentrations will be assessed in-house (Dr. Raylene Reimer’s laboratory) according to our established protocols. TNF, IL-6, IL-1, IL-8 and C-reactive protein will be quantified using Milliplex Human Cytokine kits (Millipore, Billerica, MA). Plate reading will be provided as a fee-for service through Eve Technologies Inc. (Calgary, AB).

### Sample size calculation

Sample size calculations are based on minimum participants required for a linear mixed models analysis as derived from Schmitz et al. [27]. They suggest a strong relationship between strength training and improvements in lean tissue in cancer patients. The power calculation was derived using G-Power [49,50]. The effect size of 0.63 was calculated based on relevant literature associated with similar interventions and α = 0.05 and β = 0.80 were used [22,27]. Based on these numbers, it was determined that a power of n = 32 is required for each group. Accounting for potential participant drop-out, a 20% buffer will be added and a total of n = 40 for each group, for a total n = 80. All intervention participants will be recruited for the intervention over year one of the study. Preliminary data from the EHNANCE exercise program for HN patients indicates that this recruitment goal is feasible (36 patients recruited in 4 months, with adherence at 90%).

### Analysis

Analyses will include feasibility (participants recruited; program attendance), descriptive analyses and statistical tests examining change over the program duration. The raw data will be analyzed using SPSS Version 19.0, and will primarily consist of linear mixed models. Information from the 3-Day Diet Records will be analyzed with Diet Analysis Plus 10.0 software (Wadsworth Inc.) to generate total daily energy intake, macronutrient and micronutrient intake for each participant. The resultant data will be analyzed using SPSS.

For the primary objective, we will be utilizing linear mixed models analyses, to examine change between groups and over time, with the primary outcome being body composition changes over time and between the two groups (immediate intervention and delayed intervention).

For the secondary objective, we will also examine change over time in health related fitness, QoL, functional status (Karnofsky), dietary intake, and markers of inflammation.

### Protection against bias

To protect against bias, participants will be randomly assigned to one of the two intervention arms, using randomization.com, a web-based random number generation program with a 1:1 allocation ratio. Exercise testers who will be evaluating participant fitness measures will be blinded to participant allocation. When participants are recruited, they will be told that there is an equal possibility that they will be assigned to one of the two groups (immediate lifestyle intervention or the delayed lifestyle intervention group) and that the impact of intervention timing is not yet known. Once they have completed their initial assessment, they will be contacted by the research coordinator and told which group they have been allocated and receive an explanation of the protocol and their responsibilities.

## Discussion

HN cancer survivors are at an increased risk for physical and psychological burden into survivorship, with many complications related to weight loss and muscle wasting [19,22]. This has direct implications on patient QoL, rehabilitation, and return to work [17-21]. The proposed study will provide a wide-ranging investigation into the impact of PA, diet screening and education on body composition before and following treatments. This work will specifically address whether a lifestyle approach can help to preserve muscle mass and whether there is a link between body composition and patient reported outcomes, such as QoL. The goal of this research is to address a current unmet need and respond directly to patient demand for comprehensive, patient-centered care by delivering an intervention combining PA, nutritional and educational elements. This intervention will target physical and psychosocial well-being, promoting improved management of patients’ physical and psychosocial responses to treatment as well as improving long-term outcomes. Further, this research will evaluate the comparative effectiveness of a comprehensive PA, nutrition and education intervention at different points along the cancer care trajectory, to identify the most effective clinical intervention point in patient care based on rigorously assessed health outcomes. The evidence required for successful clinical adoption and integration of this multidisciplinary clinic-based intervention will be gathered and a model will be built for future program translation. In addition, and crucial to the success of this clinic-based program, an opportunity for advanced collaboration among health care providers will be supported, ultimately enhancing the patient care experience and resulting in improved vital health outcomes.

## Abbreviations

HN: Head and neck; QOL: Quality of life; PA: Physical activity; ILI: Immediate lifestyle intervention; DLI: Delayed lifestyle intervention; DXA: Dual energy x-ray absorptiometry; PG-SGA: Patient generated – subjective global assessment tool; LSI: Leisure score index; GLTEQ: Godin leisure time exercise questionnaire; FACT-AN: Functional assessment of cancer therapy anemia; FHNSI-22: Functional assessment of cancer therapy head neck symptom index-22; TOI: Trial outcome index; CES-D: Studies on depression scale; ESAS: Edmonton symptom assessment system; KPS: Karnofsky performance score.

## Competing interests

The authors report no competing interests in this paper.

## Authors’ contributions

LC: helped to conceive of the study and participated in its design. She also drafted the manuscript. HL: participated in the development of the study design and offered clinic support to implement the study. He was also involved with editing the manuscript. RR: participated in the development of the study design and the addition of the nutritional components. She also was involved with editing the manuscript. MM: participated in the development of the study design and the physical assessment component. She also was involved with editing the manuscript. JGD: participated in the development of the study design and the addition of the depression-screening component. She also was involved with editing the manuscript.NCR: helped to conceive of the study and participated in its design. She was also involved with drafting and editing the manuscript. All authors read and approved the final manuscript.

## Pre-publication history

The pre-publication history for this paper can be accessed here:

http://www.biomedcentral.com/1471-2407/12/446/prepub
